# Clinician-Administered PTSD Scale for DSM-5,child and adolescent version: A transcultural validation

**DOI:** 10.1192/j.eurpsy.2023.1548

**Published:** 2023-07-19

**Authors:** N. Kouki, S. Bourgou, H. Rezgui, A. Naffeti, M. Hamdoun, A. Belhadj

**Affiliations:** 1 Child and adoescent psychiatry, Mongi Slim; 2 Forensic medicine, Charles Nicolle, Tunis, Tunisia

## Abstract

**Introduction:**

Posttraumatic stress disorder in the paediatric population has clinical features. The Clinician-Administered PTSD Scale for DSM-5,child and adolescent version (CAPS-CA-5) is the gold standard for the positive diagnosis.

**Objectives:**

The objectives of our work were to translate the CAPS-CA-5 into Tunisian dialectal Arabic and to validate it in our Tunisian sociocultural context.

**Methods:**

This is a descriptive cross-sectional study conducted in the child psychiatry department of Mongi Slim Hospital and the forensic medicine department of Charles-Nicolle Hospital (Tunisia), among children older than seven years who were exposed to a potentially traumatic event at least one month before. We validated the tool through translation, content, construct validity and reliability. The statistical processing for this data was carried out using SPSS 26 software.

**Results:**

We conducted our study with 150 patients. The validation was made on 146 records after the exclusion of 4 incompleted assessments.

We initially translated the CAPS-CA-5 into Tunisian dialect. We validated the content through pre-test and scientific committee evaluation.

Afterwards, we validated the construction. We calculated the Bartlett’s sphericity test (p<0.001) .The KMO index that was 0.766.

Concerning the reliability study, we found a Cronbach’s alpha coefficient equal to 0.92. We studied also the inter-raters reliability; we found an intra-class coefficient between 0.8 and 1

**Conclusions:**

We validated the first Tunisian diagnostic tool for PTSD in children according to the DSM-5 criteria with satisfactory psychometric qualities.

**Disclosure of Interest:**

None Declared

